# Styles of Coping with Stress among Healthy People and People with Diagnosis of Schizophrenia and Selected Personality Dimensions

**DOI:** 10.3390/ijerph19095129

**Published:** 2022-04-22

**Authors:** Bartosz Wiszniewski, Hanna Liberska

**Affiliations:** 1 Faculty of Psychology, Kazimierz Wielki University, 85-064 Bydgoszcz, Poland; hanna.liberska@ukw.edu.pl; 2 Multi-Specialist City Hospital of Emil Warmiński, 85-064 Bydgoszcz, Poland

**Keywords:** personality, styles of stress coping, schizophrenia

## Abstract

Background: Schizophrenia is considered a chronic and disabling mental disorder that affects approximately one percent of the world’s population. It is characterized by a variable course and its various symptoms may predominate depending on the characteristics of the person. Aim: Recognition of the personality traits and styles of stress-coping applied by healthy people and people diagnosed with schizophrenia. Methods: The study examined 60 people in total: 30 healthy people between 19 and 58 years old and29 people diagnosed with schizophrenia and 1 person with schizophrenia spectrum disorder between 25 and 72 years old. In the present study we used *Personality inventory* NEO-FFI by Paul Costa and Robert McCreae, designed to diagnose personality traits described in the five-factor model called “The Big Five” and *CISS: Coping Inventory for Stressful Situations* by N.S. Endler, J.D.A. Parker, designed to diagnose stress-coping styles.

## 1. Introduction

Schizophrenia is considered a chronic and disabling mental disorder that affects approximately one percent of the world’s population [1]. It is characterized by a set of signs and symptoms of unknown etiology, mainly defined by symptoms of observed psychosis. Contemporary specialists emphasize the usefulness of the neurodevelopmental approach to schizophrenia, which is the basis not only for more effective treatment, but also for prevention [2]. Increasing attention is also paid to the living environment, including the degree of its urbanization, social attitudes towards mental illnesses, the presence of social sources of support and their availability for people with symptoms of mental disorders [3,4,5].

According to the idea of personalized medicine, it is advisable to develop an approach to schizophrenia based on understanding and detecting individual development pathways and the risk of the occurrence of the disorder. Research on the psychological features of people showing symptoms of this psychosis can provide the basis for increasing the effectiveness and safety of personalized therapies—taking into account the psychological properties of the patient, the stages of this disorder, periods of remission, and severity of symptoms.

Among the psychological features significant for the functioning of people with somatic or mental dysfunctions, special attention is paid to personality and behavior in stressful situations.

Researchers are greatly interested not only in the issues of stress itself, but also in the ways of coping with it. The most popular concept of stress in psychology was the concept of Richard Lazarus and Suzan Folkman. The first of these two authors published a monograph in 1966 titled *Psychological stress and the coping process.* It was the first comprehensive concept of psychological stress to focus on the effects of stress and the process of fighting it. The joint work of Lazarus and Folkman resulted in a new approach to the phenomenon of stress, namely the cognitive–transactional approach, in which stress is understood as “psychological stress is a particular relationship between the person and the environment that is appraised by the person as taxing or exceeding his or her resources and endangering his or her well-being” [6]. Endler and Parker, referring to the theory of Lazarus and Folkman, presented three styles of coping with stress. They emphasized that people using different styles of coping with stress are more aware of it, as opposed to defense mechanisms, the use of which they are not aware of. According to them, style is typical behavior for an individual in various stressful situations [7]. In the cognitive–transactional approach, we talk about constantly changing cognitive and behavioral efforts to manage specific external and internal demands that are appraised as taxing or exceeding the resources of the person [8]. Overburdening the individual with requirements may lead to imbalance in the human–environment system, which in the long run results in the deterioration of mental health and the development of somatic diseases. For example, psychological burdens are much more closely related to the risk of a heart attack than factors such as smoking or cholesterol levels [9,10].

In psychology, it is emphasized that not only coping with stress, but also the interaction of the coping style with personality traits is important for the functioning and health condition of an individual [8,11]. Personality traits are part of an individual’s intrapsychic and interpersonal resources, which are important for coping with difficulties. Therefore, extraversion, classified as positive emotionality, allows you to perceive difficult situations as challenges [12,13], and optimism, according to Terelak [14,15], constitutes the so-called social competence, which increases resistance to stress. Neuroticism, as a component of negative emotionality, favorsthe perception of events as threatening and limits the effectiveness of coping with difficult situations [12,13]. There are numerous confirmations in the literature that personality plays a significant role in the way of coping with stressful situations. Many theoretical considerations and reports of empirical research confirm the thesis of treating coping with stress as a variable depending on personality predispositions [16,17,18].

In the presented research the authors rely on the concept of the “Big Five” by Paul Costa and Robert McCrae and on the proposal of styles of coping with stress by Endler and Parker. The choice of the theoretical framework was determined by theoretical and methodological considerations. Among other things, the positioning of tools in theoretical models, with relatively high methodological formalization and good psychometric characteristics of the original version, is enthusiastically assessed in critical analyses. Polish adaptations of tools that fit into the culture of the country obtained very satisfactory psychometric values [19,20,21].

People suffering from schizophrenia are characterized by a reduced ability to effectively cope with various tasks or problems, which increases the risk of them experiencing an uncomfortable state of distress. Research on the style of coping with stress and the determinants of coping with stress in the context of changes in the severity of disease symptoms provide an opportunity to identify the periods of higher effectiveness in coping with life challenges. They can also provide the basis for the temporal structure of the activity of people diagnosed with schizophrenia, increasing their chances for effective functioning and a sense of well-being.

Experiencing a stressful situation may intensify the past symptoms of the disease resulting from the psychobiological susceptibility of an individual to psychological injury, which condemns patients to experience loneliness, a feeling of being forgotten by other people (even their relatives) and even a feeling of rejection. This can change the way you perceive yourself and the world around you and generate further negative feelings, such as loss of hope for a positive future [22,23]. The mechanism responsible for the occurrence of symptoms is related to exceeding the individual’s mental endurance, which is susceptible to exogenous and endogenous loading. The accumulated physiological and psychological stress, including the exogenous burden on the life of an individual, unfavorable mental conditions, caused by the disturbance of traumatic and positive experiences and the presence of many factors that disturb biochemical and neurophysiological processes, and the effects of toxic substances and negative effects of past infections, which are intensified in adolescence, reduce the ability of maintaining homeostasis and weakens mental endurance, immunity, and leads to a global decline in intellectual functions [24]. The constellation of these factors and their interactions vary individually and over time. This creates opportunities for periodic improvement in functioning and well-being, which has a positive impact on the individual and his environment. Research on the dynamics of schizophrenia symptoms and the dissemination of their results may contribute to a change in social attitudes towards people diagnosed with schizophrenia [25,26,27]. Exogenous loading occurring during the life of an individual, an unfavorable balance of traumatic events, the multiplicity and intensity of factors disturbing the biochemical and neurophysiological processes occurring during puberty, as well as the effects of toxic substances and past infections (generally the amount of cumulative physiological stress) result in a reduction in the ability to maintain homeostasis and weakens the resources of mental endurance, resilience, and leads to global decline in intellectual functions [24,25]. The constellation of these factors and their interactions vary individually and over time. This creates opportunities for periodic improvement in functioning and the sense of well-being, which have a positive meaning for the individual and their environment. Research on the dynamics of schizophrenia symptoms and the dissemination of their results may contribute to changing social attitudes towards people diagnosed with schizophrenia [26,27,28].

There is one more very important reason for conducting research on schizophrenia, its determinants, and consequences for the individual and society. Most people trying to commit suicide have mental disorders. Research conducted in the USA shows that over 90% of suicide victims have mental disorders. Suicide attempts are most often preceded by mood disorders and psychotic disorders [29,30,31,32,33]. The World Health Organization (WHO) conducted a 5 year follow-up study of 1065 patients with psychotic disorders which showed that “the risk of suicide in schizophrenia is as great, if not greater than the risk of suicide associated with affective disorders” [31]. Contemporary scientific research shows that the lifetime suicide rate in people diagnosed with schizophrenia ranges from 4 to 13%, while the modal rate is around 10% [34].

The collected evidence shows that schizophrenia shortens life expectancy by about 10 years [35], and this is often the result of suicide. Recognition of risk factors—variables that adversely affect the course of the disease—as well as protective factors, is essential to improve the treatment of patients and to progress in the approach to reducing the frequency of decisions to take on one’s life in patients with schizophrenia. A suicide attempt can be read as a “cry for help” by a person with schizophrenia—it is a clear signal that the person is not coping with the hardships of everyday life. Psychological research indicates the importance of the personality and style of coping with stress for a person’s protective resources (or risk resources), so it is justified to recognize their role in the dynamics of schizophrenia, therapy, and the prevention of suicide attempts in people diagnosed with schizophrenia.

The aim of the presented study was to solve the problem of the frequency of choosing the style of coping with stress among healthy people and people diagnosed with schizophrenia and its dependence on the selected personality dimension.

Postawiononastępującepytaniebadawcze: Is there a significant correlation between the personality traits/dimensions and the frequency of using a specific coping style? Is this relationship present different between people diagnosed with schizophrenia and healthy people?

Therefore, the main hypothesis H1 was formulated: There is a significant correlation between the intensity of personality dimensions and the frequency of occurrence of coping styles. This relationship is different between people diagnosed with schizophrenia and healthy people.

The main hypothesis is expressed in the form of three detailed hypotheses:

**Hypotheses** **1a.** *There is a significant relationship between the intensity of the personality dimension and the frequency of using the task-focused style. This relationship is different in people diagnosed with schizophrenia and in people without schizophrenia (healthy people)*.

**Hypotheses** **1b.** *There is a significant relationship between the intensity of the personality dimension and the frequency of using the avoidance-focused style. This relationship is different in people diagnosed with schizophrenia and healthy people.*

**Hypotheses** **1c.** *There is a significant relationship between the intensity of the personality dimension and the frequency of using the emotion-focused style. This relationship is different in people diagnosed with schizophrenia and in people without diagnosed schizophrenia (healthy)*.

## 2. Material and Methods

60 adults aged 19 to 75 participated in the study. The group of healthy people (without diagnosed mental disorders) consisted of 18 women and 12 men (average age was 32). The second group consisted of 29 people diagnosed with schizophrenia and 1 person with aschizophrenia-type disorder. This group included 12 women and 18 men (the average age was 43). Both groups consisted of 26 people with vocational or technical education; 14 with secondary education; 20 with higher education. The respondents live in a Polish city with 200,000 inhabitants. All persons participating in the study were informed about the purpose of the study and their anonymity, as well as about the possibility of withdrawing from participation in the study at any time during its duration. Each of the patients agreed to participate in the study and for the researchers to use the material collected during the study.

The size of the group depended on the size of the city in which the study was conducted as well as other factors, mainly their current health condition, willingness to participate, and absence during the study.

The participants were invited to take part in the study on individually agreed dates. The research conditions were standardized. The duration of the study was adjusted to the individual needs of the participants.

The research used the CISS Coping Inventory and NEO-FFI personality inventory.

Coping Inventory for Stressful Situations (CISS) was developed by N.S. Endler; J.D.A. Parker (1990). The authors of the Polish adaptation are A. Jaworowska, J. Strelau, P. Szczepaniak and K. Wrześniewski (2009). The inventory is used to diagnose styles of coping with stress. It includes 48 statements about human behavior in stressful situations. The results are classified on three scales: task-oriented style (SSZ), emotion-oriented style (SSE), and avoidant style (SSU).

The NEO-FFI personality inventory, the authors of which are P.T. Costa, R.R. McCrae (1989), (Polish adaptation B. Zawadzki, J. Strelau, P. Szczepaniak, M. Śliwińska, 1998), is used to diagnose personality traits included in the five-factor model, defined as a model of the Big Five. It contains 60 items, twelve in each of the five scales: neuroticism, extraversion, openness to experience, agreeableness, and conscientiousness.

## 3. Results

Descriptive statistics and Spearman’s rank coefficients, and Pearson’sChi-squared test were used in the statistical analysis of the collected data. The results that met the value criteria *p* < 0.05 were considered valid.

The results presented below include the features of neuroticism and extraversion as well as the task and avoidance styles.

The features of agreeableness, openness to experience, and conscientiousness as well as the style focused on emotions were omitted later in the article due to the lack of statistical significance of the relationship between them. The results of the statistical analysis showed the irrelevance of the relationships between the style focused on emotions and personality traits both in the group of people with schizophrenia and in the control group.

Moreover, the results of the statistical analysis of the Chi-squared test did not confirm the relationship between the frequency of using the style focused on emotions and the health condition (*χ*^2^ = 0.3839; df = 2; *p* > 0.05). Therefore, Hypothesis 1c has not received the acknowledgment.

For this reason, the presentation of the results focuses on the relationships between those variables that turned out to be statistically significant, i.e., between extraversion and neuroticism, and the task-oriented and avoidant style (Table 1).

Table 2 presents descriptive statistics that show differences between the compared groups in terms of the styles of coping with stress and selected personality dimensions.

The results of the quantitative analysis indicate that the healthy people (without diagnosed of schizophrenia) obtained higher average scores on the task-oriented style scale (M = 1.97; SD = 0.61) and on the extraversion personality dimension (M = 2.23; SD = 0.679) than people with schizophrenia.The ill people (with a diagnosis of schizophrenia) obtained higher average scores on the avoidant style scale (M = 2.37; SD = 0.61) and emotion style scale (M = 2.17; SD = 0.61), and neurotic personality dimension (M = 2.37; SD = 0.77) when compared to people without symptoms of schizophrenia (Table 1).

Summarizing the above analysis, it should be concluded that healthy people are more often characterized by the task-oriented style and features of extrovert personality, while ill people more often choose the avoidant style and are characterized by a neurotic personality.

Table 2 presents the results of the analysis of the correlation of the studied variables: the style of coping with stress and personality traits. The results of statistical analysis with the use of Spearman’s rank correlation coefficients allowed us to obtain answers to the question as to whether the task-oriented style and avoidant style of coping correlate statistically significantly with neuroticism and extraversion.

Results of the analysis indicate that there is a positive correlation between the avoidant style and neuroticism (0.273; *p* < 0.05). A positive relationship between extraversion and task-oriented style was also observed (0.281; *p* < 0.05). There was also a negative correlation between the two examined personality dimensions (−0.335; *p* < 0.05).

The results of the statistical analysis confirm the Hypotheses 1a and 1b.

Next, we analyzed whether there is a significant correlation between the health conditions analyzed (among healthy people and people diagnosed with schizophrenia) and the intensity of selected personality dimensions and the frequency of stress coping styles. The Chi-squared test was used in the statistical analysis (Table 3).

The performed statistical analysis shows that the health condition of the examined person significantly differentiated the use of the avoidant style (*χ*^2^ = 18.455; df = 2; *p* < 0.05) and the severity of the dimension of neuroticism (*χ*^2^ = 10.950; df = 2; *p* < 0.05) (Table 3). In the group of healthy people, there were few results indicating a high level of avoidant style. Therefore, in the statistical analysis of the severity of the avoidance style, the subgroups with medium and high scores were combined. Among healthy people (70%), subjects were characterized by a moderate or high avoidant style, while among people diagnosed with schizophrenia this was higher (93.33%). To sum up, respondents diagnosed with schizophrenia use the avoidance style of coping with stress significantly more often than healthy people. The results concerning the dimension of neuroticism indicate that people diagnosed with schizophrenia have significantly more often a high level of neuroticism (53.33%) than healthy people (13.33%).

Statistical analysis did not show any statistically significant relationships between health conditions and the task-oriented style (*χ*^2^ = 1.127; df = 2; *p* > 0.05) or between health conditions and the intensity of the dimension of extraversion (*χ*^2^ = 0.906; df = 2; *p* > 0.05).

## 4. Discussion

The aim of this study was to identify the specific relationships between mental health, personality traits, and coping styles. The results of statistical analysis provided an answer to the question whether the relationships between personality traits and styles of relations with stress are the same or different in people diagnosed with schizophrenia and healthy people.

### 4.1. The Relationship between the Avoidance-Focused Style and the Dimension of Neuroticism

During the study, a significant positive correlation was found between the avoidant style and the dimension of neuroticism—however, the revealed correlation was of lowstrength (0.273; *p* < 0.05). The results of studies available in Polish and foreign literature [36,37,38,39] show that neurotic people prefer avoidant strategies, which is associated with a lower probability of using a problem/task-oriented strategy, and more frequent avoidance and denial. As a result, they are exposed to more stress, which results in more serious health consequences. These people are prone to negative emotions such as fear, anxiety, anger, hostility, and guilt. In difficult situations, they are characterized by avoiding thinking about a difficult situation. Generally, they try to avoid stressful situations, which can be interpreted as an escape from difficult reality. The presented research confirms this thesis.

### 4.2. The Relationship between Health Status and the Intensity of the Neuroticism Dimension

The results of the statistical analysis carried out with the Chi-squared test (*χ*^2^ = 10.950; df = 2; *p* < 0.05) showed a significant relationship between the health status of the examined person and the frequency of various levels of neuroticism. People diagnosed with schizophrenia were significantly more prone to a greater severity of neuroticism than healthy people.

The weakened psychobiological state of people diagnosed with schizophrenia, their reduced mental resistance or mental endurance, and a higher level of neuroticism—compared to people who have not been diagnosed with symptoms of mental illness—constitute a set of determinants for the preferences of an avoidant coping style in solving life difficulties. The analyses confirm this relationship. They indicate that people diagnosed with schizophrenia and high neuroticism have a stronger tendency to avoid stress than healthy people achieving low and medium levels of neuroticism. However, it should be added that in the group of healthy people, no results indicating a high level of neuroticism were recorded. At the present stage of research, there is no reason to reject the supposition that healthy people (i.e., those who have not been diagnosed with symptoms of mental disorders) who are characterized by a high level of neuroticism also prefer the avoidant style. The clarification of this issue requires the expansion of the study group.

### 4.3. The Relationship between Task-Oriented Style and Extraversion

The results of the analysis of my own research also show a positive relationship between the task-oriented style and extraversion (*r* = 0.281; *p* < 0.05). Obtained results testify that regardless of the health condition, people with high activity and the search for experience—what is characterized by extroverted individuals—are more likely to take action in stressful situations aimed at solving problems through cognitive transformations or attempts to change the situation. The results of my own research indicate that in difficult situations, certain dimensions of personality factors are conducive to more effective coping by both healthy people and by people diagnosed with schizophrenia. This provides the basis for including them among the potential group of predictors of health or protection resources. The above results confirm the results of other researchers. For example, in the study by J. Szrajda et al. [39], 58 healthy volunteers working in the fire department in the Kuyavian-Pomeranian Voivodeship were tested in order to determine the relationship (correlation) between personality and temperamental traits with manifested styles of coping with stress. The identical research tools as to the ones used in the research reported in this article were used, which gives the possibility of a more accurate comparison. The obtained results showed a positive correlation between the dimension of extraversion and the task-oriented style (*r* = 0.323; *p* < 0.05), and therefore in the studied group, a relationship of the same nature as in the discussed own research was found.

A study conducted at the University of Manitoba in Canada also compared the characteristics of Big Five with the styles of coping with stress. The study involved 298 patients with major depressive disorders. Similar to the above-mentioned studies, also in this study a significant positive correlation was found between the extraversion trait and the task-oriented style (*r* = 0.450; *p* < 0.001). The quoted study also found a significantly negative relationship between neuroticism and the task-oriented style (*r* = −0.037; *p* < 0.001), which was not found in the authors’ own research [40].

Summarizing the above considerations, it should be emphasized that both in people diagnosed with schizophrenia and in people who have not been diagnosed with mental disorders, there is a relationship between the styles of coping with stress and personality traits. At this point, it is worth emphasizing that coping with stress also depends on other factors that were not explored in this study. It is important to indicate the role of social support in active coping with stress, as well as the relationship between social support and personality [41,42].

According to researchers such as Cutron, Hessling, and Suhr (1997), personality may be associated with support because it affects the ease of establishing social contacts and determines the course of relationships in existing relationships. In addition, it determines the quality of the relationship, which affects the effectiveness of the obtained support [43]. The source of this support, in the case of many people with schizophrenia symptoms, is the family, self-help groups, and institutionalized support systems. The quality of the social support network is an indicator of the maturity and responsibility of the social environment for the condition of its members.

### 4.4. Health Status and the Frequency of Using the Avoidance-Focused Style

The results of the Chi-squared test indicate that the condition of health significantly differentiates the frequency of using the avoidant style. The literature on the subject refers ambiguously to the preference for one of the styles, although some authors point to the greater effectiveness of the task style [8]. Psychopathological symptoms may be a trigger factor in choosing the avoidant style in the case of people diagnosed with schizophrenia. Studies by other authors show that this style has a negative effect on the course of the disease. A. Kokoszka in hisresearch [37], involving 177 patients diagnosed with schizophrenia using outpatient psychiatric services, showed a moderate negative relationship between the sense of influence on the course of the disease and the style focused on avoidance, and a positive relationship between the sense of influence and the task-oriented style. Ill people choosing avoidant style have a lower sense of influence on the disease. “Patients convinced that they cannot control the source of stress (here: voice) more often“ succumb to ”the commands of the voice [41,42,43]. Where patients have a sense of control and influence, they try to solve the problem, but when the perceived source of stress is, in their opinion, beyond their influence, they use avoidance strategies” [37]. The above-mentioned results are important for clinical practice, because psychoeducation directed at people diagnosed with schizophrenia is effective when it is enriched with behavioral techniques and aimed at increasing the patient’s motivation for treatment, and the assessment of ways of coping with stress may support this process.

The above-mentioned studies [37] also indicate a negative relationship between the solution-oriented style and negative symptoms manifested in the functioning of people with diagnosed mental illness. These symptoms are manifested in social withdrawal, apathy, and emotional flatness. This makes the rehabilitation process difficult for the patient due to their difficulties in undertaking active forms of coping with the disease. Also, deepening the analysis of the dimensions of the style focused on avoidance, namely engaging in substituteactivities and looking for social contacts, could provide new clues for therapeutic work with people diagnosed with schizophrenia.

All these observations are consistent with clinical practice, therefore, taking into account the results of my own research, it is justified to recommend the inclusion of elements of stress coping training in therapeutic programs used by people working with individuals diagnosed with schizophrenia.

Due to the social importance of the problem of effective coping with difficulties by people with diagnosed mental diseases, not only is the diagnosis of schizophrenia necessary, but also to further explore the problem and define the conditions in which these people can use personal resources, resulting in an increase in their well-being.

### 4.5. Research Limitations

The size of the compared group can be seen as a factor limiting the scope of inference and generalization of research results. However, the possibilities of examining people diagnosed with schizophrenia are determined by the specificity of the disease. The study provided the similarity of the compared groups (people with a different mental health condition) in terms of their general life situation (living outside the health care facility), place, and level of education. Limitations in the selection of people were related to the current condition (remission) and motivation of people diagnosed with schizophrenia.

In the case of people diagnosed with schizophrenia, the factor limiting the focus on the task could be the duration of the study—however, the researchers decided to use these specific tools because they are tools very often used in strictly psychological studies of personality and coping style, and they have good psychometric properties. Their use in both study groups (in the group of people diagnosed with schizophrenia and in the group of people without schizophrenia symptoms) made it possible to compare the results. The results of the analysis of the material obtained with the use of the tools allowed us—which is worth emphasizing—to refer to the recognized theories of personality psychology, health, and disease psychology.

## 5. Conclusions

It was concluded that there are significant differences in personality and preferred styles of coping with stress between healthy people and those with schizophrenia. People diagnosed with schizophrenia were characterized by less adaptive personality traits and styles of coping with stress than healthy people. However, it should be noted that among people without schizophrenia symptoms, there are also people who show increased neuroticism and prefer the avoidance strategy—and among people with schizophrenia, there are those who resort to task strategies in a difficult situation. The dissemination of knowledge about the limited differences between healthy people and people with symptoms of mental illness (here: schizophrenia) regarding the relationship between coping style and personality dimensions may be the basis for changing social attitudes towards mentally ill people. In the clinical environment, the expansion of research on mental diseases and the role of psychological mechanisms governing them may allow for the personalization of the approach to patients and the improvement of therapeutic techniques, which in turn may increase the sense of psychological well-being of people suffering from mental diseases, as well as improve the psychosocial condition of their families.

## Figures and Tables

**Table 1:** The relationship between the intensity of selected personality dimensions and styles of coping with stress among healthy people and people diagnosed with schizophrenia (Spearman R-coefficient) (n = 60).

	Task-Oriented Style	Avoidant Style	Neuroticism	Extraversion
Task-oriented style	1.00	−0.079	−0.083	0.281 *
Avoidant style	−0.079	1.00	0.273 *	−0.123
Neuroticism	−0.083	0.273 *	1.00	−0.335 *
Extraversion	0.281 *	−0.127	−0.335 *	1.00

* *p* < 0.05, Source: own study.

**Table 2:** Descriptive statistics of variables: style of coping with stress and selected dimensions of personality (average values, min and max values, standard deviation).

Variables:	Valid N	Average Value	Minimal Value	Maximal Value	Standard Deviation
	People without Diagnosed of Schizophrenia				
Task-oriented style	30	1.97	1.00	3.00	0.615
Avoidant style	30	1.70	1.00	2.00	0.466
Emotion style	30	1.83	1.00	3.00	0.699
Neuroticism	30	1.77	1.00	3.00	0.679
Extraversion	30	2.23	1.00	3.00	0.679
	People diagnosed with schizophrenia				
Task-oriented style	30	1.80	1.00	3.00	0.610
Avoidant style	30	2.37	1.00	3.00	0.615
Emotion style	30	2.17	1.00	3.00	0.648
Neuroticism	30	2.37	1.00	3.00	0.765
Extraversion	30	2.07	1.00	3.00	0.691

Source: own study.

**Table 3:** The relationships between the variables: the frequency of using the coping style and personality, and the health condition of the respondents(Chi-squared).

N = 60	Healthy Individuals (N = 30)	Diagnosed Individuals (N = 30)	Sample Size	Value
**Task-oriented**				*χ*^2^ = 1127
**style**				df = 2
Low	20%	30%	15	*p* = 0.566
Medium	63.33%	60%	37	
High	16.67%	10%	8	
**Avoidant style**				χ^2^ = 18.455
				df = 2
Low	30%	6.67%	11	*p* = 0.000
Medium and high	70%	93.33%	49	
**Neuroticism**				χ^2^ = 10.950
Low	36.67%	16.67%	16	df = 2
Medium	50%	30%	24	*p* = 0.003
High	13.33%	53.33%	20	
**Extraversion**				χ^2^ = 0.906
Low	13.33%	20%	10	df = 2
Medium	50%	53.33%	31	*p* = 0.636
High	36.67%	26.67%	19	

Source: own study.

## Data Availability

The data presented in this study are available upon request from the corresponding author.

## References

[1] Jongsma H.E., Gayer-Anderson C., Lasalvia A., Quattrone D., Mulè A., Szöke A., Selten J.-P., Turner C., Arango C., Tarricone I. (2018). Treated Incidence of Psychotic Disorders in the Multinational EU-GEI Study. JAMA Psychiatry.

[2] Insel T.R. (2010). Rethinking schizophrenia. Nature.

[3] Morgan C., Lappin J., Heslin M., Donoghue K., Lomas B., Reininghaus U., Onyejiaka A., Croudace T., Jones P.B., Murray R.M. (2014). Ponownaocenadługoterminowegokursuiwynikówzaburzeńpsychotycznych: Badanie AESOP-10. Psychol. Med..

[4] Lieberman J.A., Drake R.E., Sederer L.I., Belger A., Keefe R., Perkins D., Stroup S. (2008). Science and recovery in schizophrenia. Psychiatr. Serv..

[5] Tandon R., Nasrallah H.A., Keshavan M.S. (2009). Schizophrenia, “just the facts” 4. Clinical features and conceptualization. Schizophr. Res..

[6] Lazarus S., Folkman S. (1984). Stress, Appraisal and Coping.

[7] Endler N.S., Parker L.D.A. (1990). Coping Inventory for Stressful Situations (CISS).

[8] Ogińska-Bulik N., Juczyński Z. (2010). Personality Stress and Healthy.

[9] Karasek R., Theorell T., Schwartz J.E., Schnall P.L., Pieper C.F., Michela J.L. (1988). Job characteristics in relation to the prevalence of myocardial infarction in the U.S. HES and HANES. Am. J. Public Health.

[10] Vaccarino V., Sullivan S., Hammadah M., Wilmot K., Al Mheid I., Ramadan R., Elon L., Pimple P., Garcia E., Nye J. (2018). Mental Stress-Induced-Myocardial Ischemia In Young Patients with Recent Myocardial Infarction. Circulation.

[11] Hulbert C.A., Jackson H.J., Mc Gorry P. (1996). Relationship between personality and course and outcome in early psychosis: A review of the literature. Clin. Psychol. Rev..

[12] Dolińska-Zygmunt G. (1996). Elements of Health Psychology.

[13] Macia P., Gorbena S., Gomez A., Barranco M., Iraurgia L. (2020). Role of neuroticism and extraversion in the emotional health of people with cancer. Heliyon.

[14] Terelak J. (2001). Psychological Stress.

[15] Terelak J. (2017). Life Stress: A Psychological Perspective.

[16] Olszewska S., Spryszyńska M. (2013). Personality and Styles of Coping with Stress of Security Service Managers. Opusc. Sociol..

[17] Oehler A., Wendt S., Wedlich F., Horn M. (2018). Investors’ personality influences Investment Decisions: Experimental Evidence on Extraversion and Neuroticism. J. Behav. Financ..

[18] Nicholson N., Nicholson N., Creevy M.F., Soane E., Willman P. (2001). Risk Propensity and Personality.

[19] Hogan R., Conely J.C., Kramer J.J. (1991). Review of The Neo Personality Inventory. The Tenth Mental Measurement Yearbook.

[20] Leong F.T.L., Dollinger S.J. (1991). NEO Personality Inventory. Test Critiques.

[21] Tinsley H.E.A. (1994). NEO-Personality Inventory—Revised. Test Critiques.

[22] Sawicka S. (2005). Comparision of the doping styles among schizophrenic patients, dependent patients and patients with a dual diagnosis. Psychiatr. Pol..

[23] Steuden S. (1997). Dynamics of Personality Changes in People Diagnosed with Schizophrenia.

[24] Borkowska A., Rybakowski J. (1997). The Possibility of Improving Cognitive Functions in Patients with Schizophrenia.

[25] Gonzalez-Blanko L., Garcia-Portilla M.P., Dal Santos F., Garcia-Alvarez L., Fuente-Tomas L., Menendez-Miranda I., Bobes-Bascaran T., Saiz P., Bobes J. (2019). Predicting real-world functioning in outpatients with schizophrenia: Role of inflammation and psychopathology. Psychiatry Res..

[26] McCutcheon R., Marques T., Howes O. (2019). Schizophrenia—AnOverview. JAMA Psychiatry.

[27] Green M.F., Horan W.P., Lee J. (2019). Nonsocial and social cognition in schizophrenia: Current evidence and future directions. World Psychiatry.

[28] Barch D.M. (2019). Nonsocial and social cognitive function in psychosis: Interrelationships, specificity and innovative approaches. World Psychiatry.

[29] Hawton K., van Heeringen K. (2009). Suicide. Lancet.

[30] Mann J.J. (2002). A current perspective of suicide and attempted suicide. Ann. Intern. Med..

[31] Sher L., Oquendo M.A., Mann J.J. (2001). Risk of suicide in mood disorders. Clin. Neurosci. Res..

[32] Bradvik L. (2018). Suicide risk and mental disorders. Int. J. Environ. Res. Public Health.

[33] Sartorius N., Jablensky A., Korten A., Ernberg G., Anker M., Cooper J.E., Day R. (1986). Early manifestations and first-contact incidence of schizophrenia in different cultures. A preliminary report on the initial evaluation phase of the WHO Collaborative Study on determinants of outcome of severe mental disorders. Psychol. Med..

[34] Siris S.G. (2001). Suicide and schizophrenia. J. Psychopharmacol..

[35] White J., Gray R., Jones M. (2009). The development of the serious mental illness physical Health Improvement Profile. J. Psychiatr. Ment. Health Nurs..

[36] Kokoszka A. (2008). Perception of self-influence on the course of schizophrenia and adherence to antipsychotics. Eur. Neuropsychopharmacol..

[37] Gawęda Ł., Buciński P., Staniszewski K., Słodki Z., Sym A., Kokoszka A. (2008). Relationships between insight, self-influence on illness course, doping styles and psychopathology in schizophrenia. Psychiatria.

[38] Gunther K.C., Cohen L.H., Armelia S. (1999). The role of neuroticism in daily stress and coping. J. Personal. Soc..

[39] Szrajda J., Tudorowska M., Kujawski S., Weber-Rajek M., Sygit-Kowalkowska E., Kobos Z., Słomko J., Tafil-Klawe M., Zalewski P. (2017). The Big Five Personality and Temperament Traits and Their Correlation with Styles of Coping with Stress in the Fire Brigade Officers. J. Educ. Cult. Soc..

[40] McWilliams L., Cox B., Enns M. (2003). Use of the Coping Inventory for Stressful Situations in a Clinically Depressed Sample: Factory Structure, Personality Correlates, and Prediction of Distress. J. Clin. Psychol..

[41] Siudem A. (2008). Styles of Coping with Stress and Personality.

[42] De Almeida L., Carrer M., De Souza J., Pillon S. (2018). Evaluation of social suport and stress in nursing students. Scielo-Sci. Electron. Libr. Online.

[43] Sęk H., Cieślak R. (2004). Social Support for Stress and Health.

